# Cutaneous Malignant Melanoma Metastatic to the Larynx and Trachea: A Case Report and Review of the Literature

**DOI:** 10.3390/life13071452

**Published:** 2023-06-27

**Authors:** Mircea Sorin Ciolofan, Carmen Aurelia Mogoantă, Iulică Ioniță, Mihaela Roxana Mitroi, George F. Mitroi, Florin Anghelina, Alexandru Nicolae Vlăescu, Alina Nicoleta Căpitănescu, Alina Maria Vîlcea, George G. Mitroi, Oana Maria Ică, Loredana Elena Stoica

**Affiliations:** 1 Department of Otorhinolaryngology, Faculty of Medicine, University of Medicine and Pharmacy of Craiova, 200349 Craiova, Romania; 2 Department of Urology, Faculty of Medicine, University of Medicine and Pharmacy of Craiova, 200349 Craiova, Romania; 3 Dr. Vlăescu Central Polyclinic, Department of Otorhinolaryngology, 200638 Craiova, Romania; 4 Department of Dermatology, Faculty of Medicine, University of Medicine and Pharmacy of Craiova, 200349 Craiova, Romania

**Keywords:** melanoma, metastasis, larynx, immunohistochemistry, mucosal melanoma metastasis

## Abstract

Malignant melanoma rarely develops in mucous membranes. Statistical data show that approximately 0.6–9.3% of patients with cutaneous malignant melanoma will develop metastases in the upper aerodigestive tract mucosa, and within these metastatic sites, the least common are the laryngeal and tracheobronchial ones. This exceedingly rare clinical entity has no clear treatment recommendations; radical surgery does not seem to benefit the patient in term of life expectancy. We present the case of a 56-year-old male patient diagnosed with laryngeal and tracheobronchial melanoma metastases. Prior to admission to our clinic the patient had a personal history of malignant melanoma of the nuchal region operated on 7 years ago, malignant melanoma of the gallbladder and metastatic left axillary polyadenopathy for which he underwent surgical treatment 3 months prior. Histopathological and immunohistochemical reports established the diagnosis of laryngeal metastasis of malignant melanoma. Genetic molecular analysis was positive for B-Raf (BRAF) gene and hence Vemurafenib was administered, with a favorable outcome at the one-year follow-up. Nevertheless, there are currently no clear universally accepted guidelines for the treatment of laryngeal melanoma, mainly due to the rarity of this clinical entity. We conducted a review of similar cases reported in the literature. Interestingly, reviewing the cases reported in the literature, it appears that laryngeal metastases of a primary cutaneous melanoma are more common in men, with an average time to metastasis of 4.3 years.

## 1. Introduction

Up to 95% of the malignant tumors of the larynx are squamous cell carcinomas, followed by adenocarcinomas or glandular tumors, which account for up to 1%, and less common sarcomas, melanomas, and other rare tumors. Mucosal melanomas of the head and neck are a group of uncommon malignancies, which exhibit an aggressive pattern of evolution and poor prognosis. The most common sites for malignant mucosal melanomas within the head and neck region are the nasal cavities, the paranasal sinuses, followed by the oropharyngeal region, the larynx and esophagus [1,2]. Approximately 0.6–9.3% of patients with cutaneous melanoma will develop metastases to the mucosa of the upper aero or digestive tract, and 12% of those metastatic sites will be to the larynx [3]. Many new treatment options have emerged over the years, in particular targeted therapies, such as receptor tyrosine kinase (c-KIT) inhibitors, neuroblastoma RAS viral oncogene homolog (NRAS/MEK) or BRAF-targeted therapies as well as immunotherapies, such as anti-cytotoxic T-lymphocyte-associated protein-4 (CTLA-4) and anti-programmed cell death protein 1 (PD-1/PD-L1) antibodies, have changed the course of cutaneous melanoma management [4]. Although most patients may have multiple metastatic sites upon admission, these lesions can be challenging to diagnose. Limited clinical data are available for cutaneous melanoma metastatic to the larynx. Searching through the PubMed database using ”melanoma” in different syntaxes reveals over 1500 articles, of which very few address the subject of laryngeal metastases of a primary cutaneous melanoma. Moreover, most such articles were not eligible for analysis in our study, due to the fact that they referred either to the wrong disease, to primary mucosal melanoma or to laryngeal metastases of another condition. We report a case of metastatic melanoma to the larynx. Additionally, we summarize the available literature describing this particular clinical evolution pattern of cutaneous malignant melanoma. 

## 2. Case Report

A 56-year-old male patient presented to our otorhinolaryngology clinic with ongoing hoarseness over the past two months. He had a medical history of malignant melanoma of the skin in the nuchal region for which he underwent surgery and oncological treatment with Nivolumab seven years prior. Moreover, the patient underwent cholecystectomy approximately 3 months prior, with a histological result of undifferentiated malignant tumor, infiltrating the gallbladder wall and mucosa, as well as axillary lymphadenectomy, with a histological result of undifferentiated malignant infiltration within the excised lymph node. The diagnosis at that time was undifferentiated malignant proliferation since melanoma mutation status was not evaluated. The resection pieces from the gallbladder and lymph node were not subjected to immunohistochemical tests. 

**Figure 1:**
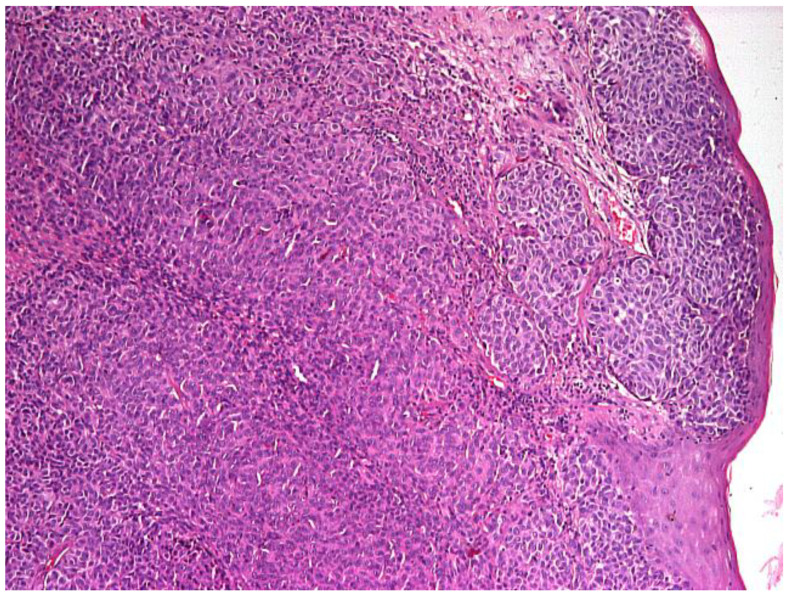
Islands of malignant cells with round or oval nuclei, with anisocoria and hyperchromasia, with infiltration of the vocal cord mucosa; hematoxylin eosin (HE) staining, optical microscopy, magnification 40×.

**Figure 2:**
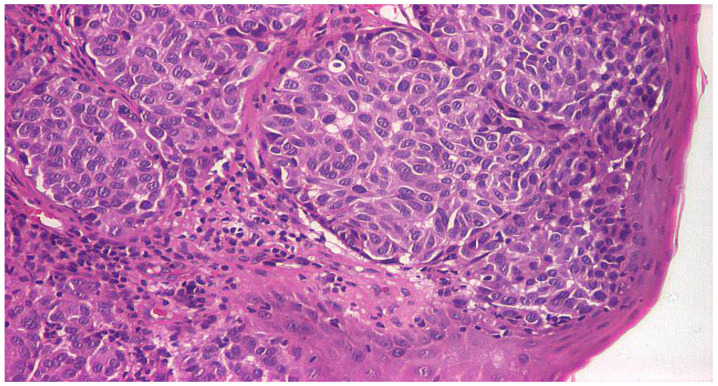
Islands of malignant cells with round or oval nuclei, with anisocoria and hyperchromasia, with infiltration of the vocal cord mucosa; hematoxylin eosin (HE) staining, optical microscopy, magnification 100×.

Upon admission to our clinic, the patient had no other comorbidities, and his general condition was good, vital signs within physiological limits and routine bloodwork within normal parameters. Upon clinical examination, indirect laryngoscopy revealed a tumoral mass located on the right vocal fold. Given the rarity of laryngeal melanoma, the presumptive diagnosis was that of an undifferentiated tumor with clear signs of malignant proliferation. Through microlaryngoscopy, under general anesthesia, right vocal fold type I cordectomy was performed the following day, without the possibility of ensuring negative excision margins, with a favorable postoperative evolution. The excised lesion of the vocal fold was forwarded to the histopathology department and the result confirmed our presumptive diagnosis, with the report describing areas of laryngeal mucosa showing undifferentiated malignant proliferation within the laryngeal corion (Figure 1 and Figure 2). 

**Figure 3:**
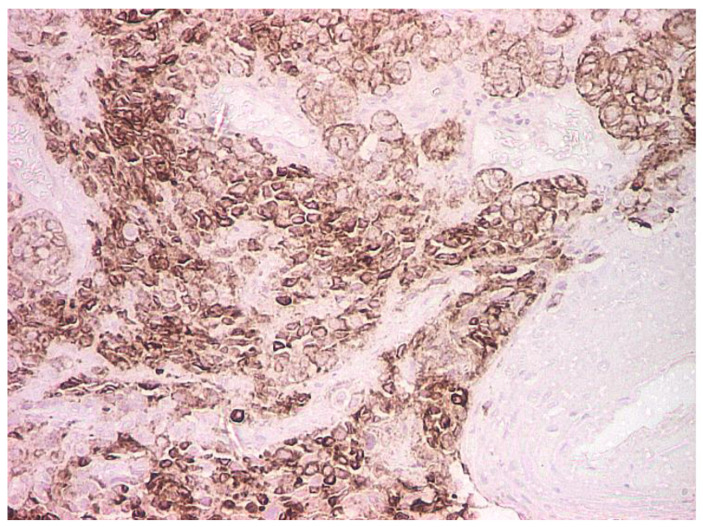
HMB- 45 melanocytic marker, positive in the tumor cells and negative in the remaining epithelium of the vocal cord; immunohistochemistry (IHC), magnification 100×.

**Figure 4:**
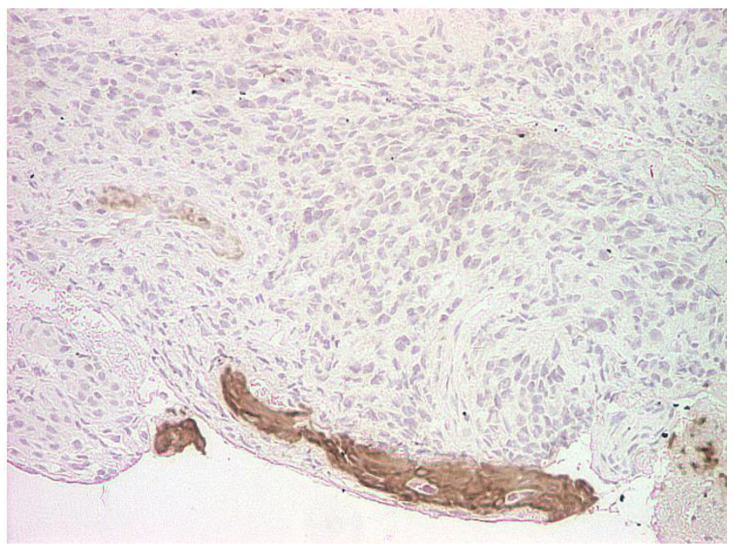
AE1-AE3 epithelial marker, negative in the tumor cells, but positive in the remaining epithelium of the vocal cord; IHC, magnification 100×.

**Figure 5:**
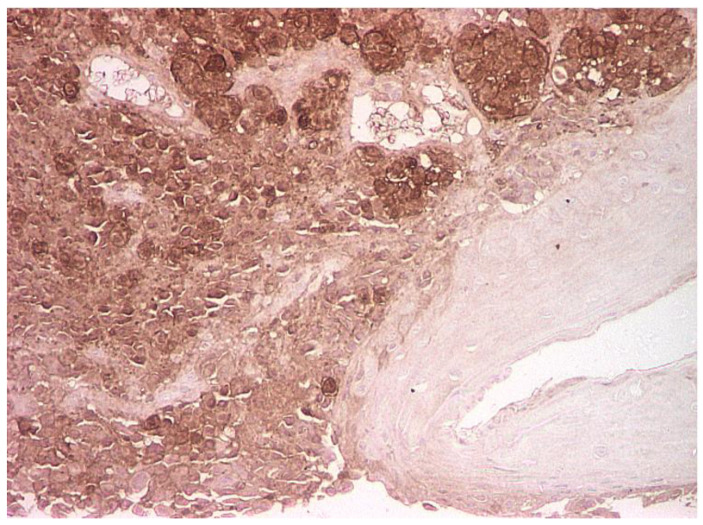
S100 melanocytic marker, positive in tumor cells and negative in the remaining vocal cord epithelium; IHC, magnification 100×.

**Figure 6:**
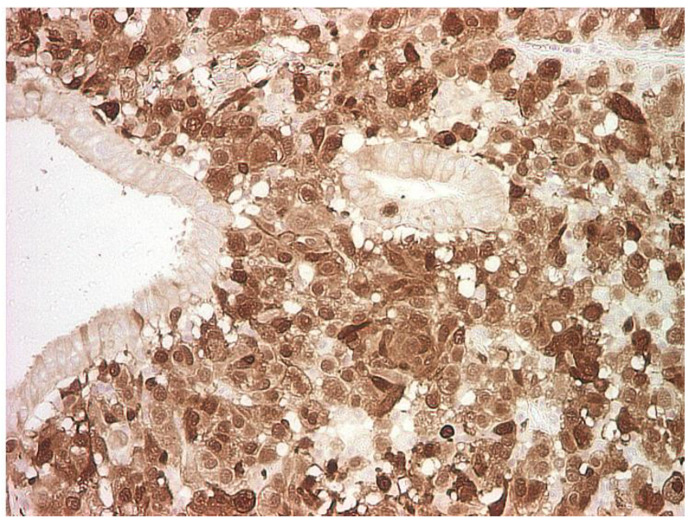
S100 melanocytic marker, positive in tumor cells within the paraffin-embedded block of tissue of the gallbladder; IHC, magnification 100×.

The personal history of cutaneous melanoma as well as malignant proliferations in the gallbladder and axillary lymph nodes raised the suspicion of melanoma with multiple metastases, including laryngeal. Thus, all the paraffin-embedded blocks of tissues, including those from the gallbladder and axillary node, were subjected to immunohistochemical testing. Immunohistochemical findings confirmed our suspicion; the report described undifferentiated malignant proliferation infiltrating the right vocal fold, gallbladder and the lymph node, sharing common cytomorphological traits: negative for cytokeratins (MNF116, general Broad spectrum cytokeratin marker), lymphoid markers (B-lymphocyte antigen CD20), and positive for Vimentin (VIM), S100, HMB45 and Melan-A antigen (Figure 3). The final diagnosis of certainty was melanoma with multiple metastases at the larynx, gallbladder and lymph nodes. Further immunohistochemical examination of the right vocal fold revealed AE1/AE3 negativity within the tumoral cells and intense positivity and diffusivity within the stratified squamous epithelial tissue (Figure 4). S100 was intensely positive and diffuse within the tumoral cells (Figure 5), and also within the gallbladder cells (Figure 6). The patient was referred to the oncology department where he underwent genetic molecular analysis from the paraffin-embedded block of tissue, the right vocal fold lesion.

**Figure 7:**
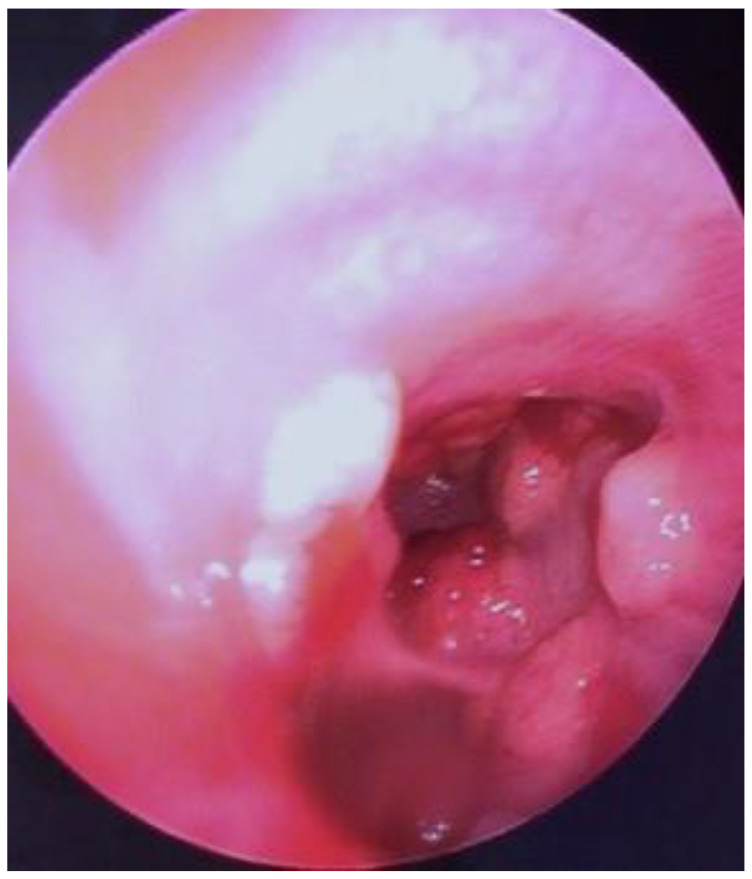
Exploratory tracheobronchoscopy revealing multiple endotracheal- and endobronchial-spouted findings.

Pending the genetic molecular report, one month later, the patient was readmitted for moderate dyspnea, recurrent haemoptysis, dysphonia and asthenia. Pulmonary radiography (chest X-ray) revealed hilar enlargement, the accentuation of bilateral interstitial markings around and beneath the hilum. Further spirometry investigation revealed moderate obstructive ventilatory impairment, with a forced expiratory volume (FEV) of 50%. Exploratory tracheobronchoscopy was performed, revealing multiple endotracheal- and endobronchial-spouted determinations within the trachea and the left main bronchia, raising the suspicion of tracheal metastatic melanoma, which is also a rare clinical entity (Figure 7). Biopsy was not repeated, given the already known diagnosis of the patient, the clinical context and the hemorrhagic risk of the procedure.

The genetic molecular analysis from the paraffin-embedded block of tissue (right vocal fold lesion), revealed a p.V600E mutation within the BRAF gene. The patient was administered Vemurafenib alongside immunotherapy with Ipilimumab and Nivolumab. Patient follow-up was favorable and uneventful for more than a year despite the advanced state of the disease. This goes to show the importance of genetic analysis which leads to an increase in the survival rate.

## 3. Discussion

Cutaneous melanoma (CM) is widely regarded as the most dangerous form of skin tumor, responsible for up to 90% of deaths caused by skin cancers. Epidemiological studies have shown a considerable increase in the incidence of melanoma, especially in male patients over 60 years old [5].

Melanomas represent malignant tumors that emerge from melanocytes and pigment cells. These tumors may bloom from either stem melanocytes bearing certain cytogenetic variations or form mature melanocytes expressing secondary cytogenetic alterations owing to external stimuli [6,7]. Melanocyte precursors traverse from the neural crest toward their final stop, passing through the embryonic mesenchyme, across distinct pathways, the majority of them winding up in the skin epidermis and dermis; meanwhile, some may end up in other sites, like the mucosal membranes belonging to the respiratory tract, the gastrointestinal tract or the genitourinary tract [4,6,8]. Of these sites, occurrence in the genitourinary tract is extremely rare, with only a total of 147 cases identified in a recent systematic review [9].

Mucosal melanomas of the head and neck were first described by Weber et al. in 1856 and have since been the subject of numerous retrospective studies [10]. Approximately 2.2–2.6 million new cases of mucosal melanoma are diagnosed annually, half of which appear in the upper region of the aerodigestive tract [11,12,13,14]. 

Limited clinical data are available for CM metastatic to the larynx. Searching through the PubMed database using ”melanoma” in different syntaxes reveals over 1500 articles, of which very few address the subject of laryngeal metastases of a primary cutaneous melanoma, which proves the rarity of this clinical evolution pattern. Exact syntaxes used for database query were as follows: ”larynx melanoma metastases”, ”laryngeal melanoma metastases”, ”neck melanoma”, ”neck melanoma metastases”, ”aerodigestive melanoma”. The search was made for articles published between 2014 and April 2023, since the last systematic review on this topic was carried out in 2014. Most articles identified after the search were not eligible for analysis, due to the fact that they referred either to the wrong disease, to primary mucosal melanoma or to laryngeal metastases of another condition. To our knowledge, this is the first article on this topic after Arabi Mianroodi et al. reported a similar case in 2013 and Santos RS et al. documented a case series of four patients in 2016 [15,16].

Melanomas can metastasize either by the lymphatic or hematogenous route, usually into the nearest lymph nodes depending on the location of the primary tumor. However, there is currently no evidence that this principle of proximity for lymphatic metastases also applies to organ metastases; thus, melanoma metastases can be located anywhere in the body. Distant metastases have a very poor prognosis [17]. Studies show that approximately 90% of patients who benefit from surgical treatment for cutaneous melanoma will be cured; however, 5% of these patients will develop metastatic melanoma which will lead to death within 10 years, despite the lack of metastases at the time of initial diagnosis [18]. The 10-year life expectancy for this category of patients who develop melanoma metastases is 4.5–8% [19]. Regarding the prognosis of the disease depending on the location of the primary tumor, studies show the risk of death is approximately twice as high in patients with melanoma of the head or neck than in patients with other locations of the primary tumor, as seen in our case, our patient having the primary tumor at the level of the nuchal region [20]. However, these findings are contradicted in the article by Lideikaitė et al. in which the 10-year survival rates for the head and neck regions were the second lowest, the first being tumors located in the back and breasts [21].

Regarding the upper aerodigestive tract, Mifsud et al. reported the palatine tonsils as the most common site for melanoma metastases, consistent with a previous report by Henderson et al. [3,22]. However, these are the only systematic reviews that we could identify in the PubMed database, both being relatively old, from 2014 and 1986, respectively. Contrary to these papers, one study reported the nose and paranasal sinuses as the most common site, followed by the oral cavity [23]. Moreover, the first two articles present 91 patients (37 and 54, respectively), whereas the third article only presents 17 patients [3,22,23]. In our opinion, the contradictory results as well as the small number of patients included in these studies represent insufficient data to be able to establish a clear site prone to metastasis. 

Mucosal melanomas show a more aggressive behavior when compared to skin melanomas and are more inclined to metastasize or recur in local, regional and distant sites, resulting in a high rate of cause-specific death. The prognosis is oftentimes grim, most published reports documenting dismal 5-year survival rates. Malignant melanoma metastatic to the mucosa is especially rare. Reviewing specialized literature shows that approximately 0.6–9.3% of patients with cutaneous melanoma will develop metastases to the mucosa of the upper aero or digestive tract, and 12% of those metastatic sites will be to the larynx [3]. There are only a small number of reports regarding intratracheal metastasis [24,25,26,27,28]. Endobronchial metastasis from melanoma is uncommon [29]. 

The biopsy of the lesions was followed by histopathologic and IHC examination to establish a diagnosis. Melanoma has a wide spectrum of histologic features which mimic epithelial, hematologic, mesenchymal, and neural tumors. In our case, the histopathological diagnosis was an undifferentiated malignant proliferation. Immunohistochemistry has been the primary tool to distinguish melanomas from these other tumors. Malignant melanomas varyingly express S-100 protein and melanocytic markers, comprising MART-1/Melan-A, HMB-45, tyrosinase and of MITF. While S-100 has a greater sensitivity, HMB-45 is considered more specific [25,30]. The scarcity or lack of melanin makes for a difficult diagnosis, one that requires immunohistochemical techniques. Amelanotic melanoma cells test positive for S-100, Melan-A, MITF, HMB-45, Vimentin, and test negative for cytokeratin [31]. 

The diagnosis depends on histopathologic evaluation and appearance and also immunoreactivity with S100 protein and melanocytic markers including HMB-45, Melan-A, or PNL-2 [32]. The presence of S100 protein and reactivity for any one of the foregoing melanocytic markers in a pleomorphic epithelioid or spindle cell neoplasm is almost diagnostic of melanoma [33]. Increased attention is conveyed to the high prevalence of activating mutations within the c-KIT oncogene (CD117), which is encountered in 80% of primary mucosal melanomas, which, on the other hand, have little to no pathogenic relevance in cutaneous melanomas [6,8,34,35]. Mutations in the BRAF gene, encountered in almost 70% of all cutaneous melanomas, were present in fewer than 10% of primary mucosal melanomas [6,8,35,36]. Among the activating BRAF mutations associated with tumors, the most frequent is V600E. The mutation predisposes to inhibiting apoptosis, increasing tumor invasiveness and represents an early event in the course of carcinogenesis. V600E is associated with a high-risk form of the disease (early onset, primary damage to the trunk, lack of chronic skin damage, reduced survival), hence the importance of therapeutic inhibition of the mutant BRAF protein. In 2011, the United States Food and Drug Administration (FDA) approved the drug Vemurafenib—a selective oral inhibitor of the kinase activity of BRAF that contains the V600E mutation—as a treatment in unresectable or metastatic malignant melanoma [37]. 

Current guidelines of mucosal melanoma management recommend the use of BRAF-targeted agents such as Vemurafenib as a first-line treatment for advanced metastatic disease [38], although the combination therapy of Ipilimumab (anti-cytotoxic T-lymphocyte-associated protein-4 antibody) and Nivolumab (anti-programmed cell death protein 1 antibody) also demonstrated favorable results in terms of five-year survival rates [39]. This combination is approved by the FDA for patients harboring the BRAF mutation and unresectable melanoma or metastatic melanoma. Nevertheless, this regime also carries a higher risk of adverse effects, up to 59% [40]. Other administration regimens of three or more drugs combined are currently undergoing clinical trials, with promising results [41]. Moreover, a combination of immunotherapy and chemotherapy, biochemotherapy, has previously showed considerable response rates and a favorable effect on a progression-free interval but with no impact on overall survival [11,42]. However, one recent study demonstrated that even these new therapeutic approaches present limits due to the acquisition of treatment resistance [43].

Selective inhibitors for targeted therapy have been sanctioned since 2011, including BRAF inhibitors, such as Vemurafenib or Dabrafenib, MEK inhibitors, like Trametinib or Binimetinib, as well as c-KIT inhibitors, providing an appealing opportunity to advance adjuvant therapies targeting head and neck mucosal melanomas (HNMM), particularly for patients displaying advanced stages of the disease, both locoregional or metastatic. Patients with BRAF V600 mutations of unresectable or metastatic melanomas, may benefit from Vemurafenib, Dabrafenib or Trametinib, usually in combined therapy. Malignant cells with V600E mutation proliferate independently from the growth factor. Recent studies have shown that V600E is associated with high-risk forms of the disease (early onset, primary damage to the trunk, lack of chronic skin damage, reduced survival), hence the importance of the therapeutic inhibition of the mutant BRAF protein. The mutation predisposes towards an inhibition of apoptosis and an increase in tumor invasiveness; it is identified in approximately 50% of malignant melanoma cases [4,6]. Radiotherapy can also be used for a better locoregional control after surgery, but it does not prevent recurrences at a distant site [23]. Regardless of treatment, overall survival in MMHN is poor and rarely is it the case that 5-year survival rates exceed 30%. It is generally agreed upon that radical surgical resection of the primary tumor offers the best chance of local control and cure, whereas the role of elective neck resection and adjuvant-postoperative radiotherapy remains unclear [11,35].

To date, relatively few cases of primary cutaneous melanoma with laryngeal metastases have been reported. The characteristics, clinical evolution and therapeutic approach to these patients are presented in Table 1. The mean patient age was 56-years-old and average time period to larynx metastasis was 4.3 years. Interestingly, most patients were men (19 out of 24 patients, 79.16%), while women represented only 20.84% of patients (5 out of 24 cases) [15,16,33,44,45,46,47,48,49,50,51,52,53,54,55,56,57].

## 4. Conclusions

Mucosal melanomas are aggressive tumors with a poor prognosis in malignant melanoma metastatic to the larynx, trachea or bronchi. Taking into consideration the low survival rate, medical teams should focus on treatment plans centered on the patient’s quality of life. New treatments such as immunotherapies or targeted therapies can change the prognosis, and they may offer interesting new perspectives. 

## Figures and Tables

**Table 1:** Summary of primary cutaneous melanoma with laryngeal metastases cases.

Article	Age/Sex	Primary Cutaneous Melanoma Site	Time Interval until Laryngeal Metastasis (Years)	Metastases Location (Larynx Involvement)	Other Metastatic Sites	Clinical Approach
Arabi Mianroodi A et al. [15]	62/M	Left tragus	1.5	Left laryngeal ventricle	Brain (frontal lobe)	Surgical excision
Santos RS et al. [16]	39/M	Axilla	4	n/a	Lung, liver, pericardum	Chemotherapy
Lanson BG et al. [33]	61/M	Left supraclavicular skin	7	Supraglottic posterior commisure region	Lung, bone	Surgical excision, radiotherapy
Massie F et al. [44]	52/M	n/a	n/a	Subglottis	n/a	n/a
Massie F et al. [44]	49/M	n/a	n/a	Epiglottis, true and false vocal cords	n/a	n/a
Massie F et al. [44]	50/M	n/a	n/a	Epiglottis	n/a	Surgical excision
Massie F et al. [44]	78/M	n/a	n/a	Right aryepiglottic fold	n/a	Surgical excision
Massie F et al. [44]	48/M	n/a	n/a	Right hemilarynx	n/a	Surgical excision
Fisher GE et al. [45]	63/F	Right shoulder	3	Right vocal cord		Surgical excision of the right true and false cords
Loughead JR et al. [46]	68/M	Right axilla	Metastases and the primary tumor were diagnosed simultaneously	Left vocal cord	n/a	Surgical excision
Faaborg-Andersen K et al. [47]	46/F	Back	2	Left arytenoid	n/a	Radiotherapy
Franzoni M et al. [48]	59/M	n/a	n/a	Epiglottis, left false cord	n/a	Total laryngectomy, radiotherapy
Franzoni M et al. [48]	72/M	n/a	n/a	Epiglottis	n/a	Total laryngectomy
Auriol M et al. [49]	38/M	n/a	n/a	Epiglottis, aryepiglottic fold, hypopharynx	n/a	n/a
Bauer J et al. [50]	70/M	n/a	8	Right true and false vocal cords	n/a	Radiotherapy
Chamberlain D et al. [51]	55/M	Chest	14	Right arytenoids, right true and false vocal cords	n/a	Radiotherapy
Freeland AP et al. [52]	43/F	n/a	n/a	Subglottis, left aryepiglottic fold, epiglottis	n/a	n/a
Ferlito A et al. [53]	75/F	Right cheek	3	Left half of the laryngeal wall of the epiglottis, extending to the pharyngeal wall and ventricular fold	n/a	Chemotherapy, radiotherapy
Ferlito A et al. [53]	60/M	Medium third of the right leg	Less than 1 year (10 months)	Laryngeal wall of the epiglottis and the right ventricular fold	n/a	Chemotherapy
Morgan AH et al. [54]	42/M	n/a	2.5	Both false cords	n/a	Carbon dioxide LASER excision
Morgan AH et al. [54]	43/M	n/a	5	Subglottis	n/a	Carbon dioxide LASER excision
Ikeda M et al. [55]	42/M	Left lower leg	2	Left laryngeal surface of epiglottis	Brain, liver, lung	Surgical excision, lasertherapy
Pau H et al. [56]	78/F	Right calf	4	Left aryepiglottic fold	n/a	Surgical excision
Koltsidopoulos P et al. [57]	55/M	n/a	n/a	Right aryepiglottic fold	n/a	Surgical excision, chemotherapy, radiotherapy

## Abbreviations

BRAF	B-Raf
c-KIT	Receptor tyrosine kinase
HE	Hematoxylin eosin
NRAS/MEK	Neuroblastoma ras viral oncogene homolog
CTLA-4	Anti-cytotoxic T-lymphocyte-associated protein-4
PD-1/PD-L1	Anti-programmed cell death protein 1
VIM	Vimentin
IHC	Immunohistochemistry
FEV	Forced expiratory volume
CM	Cutaneos melanoma
HNMM	Head and neck mucosal melanoma
FDA	Food and Drug Administration
